# Effects of intermittent theta-burst transcranial magnetic stimulation on cognition and hippocampal volumes in bipolar depression

**DOI:** 10.1080/19585969.2023.2186189

**Published:** 2023-03-16

**Authors:** Ivan J. Torres, Ruiyang Ge, Alexander McGirr, Fidel Vila-Rodriguez, Sharon Ahn, Jayasree Basivireddy, Nazlin Walji, Sophia Frangou, Raymond W. Lam, Lakshmi N. Yatham

**Affiliations:** a Mood Disorders Centre of Excellence, Department of Psychiatry, University of British Columbia, Vancouver, British Columbia, Canada; b British Columbia Mental Health and Substance Use Services, Vancouver, British Columbia, Canada; c Department of Psychiatry, University of Calgary, Calgary, Alberta, Canada; d Hotchkiss Brain Institute, University of Calgary, Calgary, Alberta, Canada; e Non-Invasive Neurostimulation Therapies Laboratory, University of British Columbia, Vancouver, British Columbia, Canada

**Keywords:** Bipolar disorder, cognitive impairment, memory, rTMS, theta-burst, cognition

## Abstract

**Introduction:**

Repetitive transcranial magnetic stimulation (TMS) is increasingly used to treat neurocognitive symptoms in mood disorders. Intermittent theta burst stimulation (iTBS) is a brief version of TMS that may preferentially target cognitive functions. This study evaluated whether iTBS leads to cognitive improvements and associated increased hippocampal volumes in bipolar depression.

**Methods:**

In a two-site double-blind randomised sham controlled trial (NCT02749006), 16 patients received active iTBS to the Left Dorsolateral Prefrontal Cortex (DLPF) and 15 patients received sham stimulation across four weeks. A composite neuropsychological score and declarative memory scores served as the cognitive outcomes. Hippocampal volumes were derived from T1 weighted MRI scans using the longitudinal ComBat method to harmonise data across sites.

**Results:**

No significant improvements were observed in any cognitive variables in the active relative to the sham group; however, there was a trend for increased left hippocampal volume in the former. Left hippocampal volume increases were associated with improvements in nonverbal memory in the active group.

**Conclusions:**

Although cognitive improvements were not associated with iTBS, the finding that hippocampal volume increases were associated with memory improvement suggests there may be some level of prefrontal-temporal neuroplasticity that could support cognitive change in future studies of iTBS in bipolar disorder.

## Introduction

Repetitive transcranial magnetic stimulation (TMS) is commonly used to treat depressive symptoms, but its role in cognitive enhancement is increasingly recognised in both healthy and clinical populations (Martin et al. 2017; Jiang et al. 2019; Begemann et al. 2020). Not surprisingly, TMS has been applied to primary cognitive disorders and dementias as a tool to assist with diagnosis, prediction/prognosis, and treatment of cognitive impairments associated with these illnesses (Di Lazzaro et al. 2021). Given the recognition that cognitive impairments are also highly prevalent among a wide range of psychiatric illnesses (Abramovitch et al. 2021), the use of non-invasive brain stimulation techniques has major relevance to many psychiatric populations. One of these illnesses is bipolar disorder, which is associated with cognitive impairments (Torres et al. 2007) that have a detrimental impact on daily functioning (Depp et al. 2012; Tse et al. 2014).

Theta-burst stimulation (TBS) is a variant of TMS which achieves short-term facilitation and inhibition with shorter stimulation protocols (Huang et al. 2005). Intermittent TBS (iTBS) is delivered in short trains, typically of 2 s duration every 10 s, and due to its facilitatory effect, iTBS exhibits strong cognitive enhancing potential (Demeter 2016; Ngetich et al. 2020).

Given the extensive involvement of the dorsolateral prefrontal cortex (DLPFC) and related brain networks in a range of cognitive abilities, prior studies investigating pro-cognitive effects of TBS have frequently targeted this region (Ngetich et al. 2020). However, few studies have evaluated the potential cognitive benefits following multi-session iTBS treatments in clinical populations (Demeter 2016). Our group and others observed left DLPFC iTBS related cognitive improvements in patients with major depression (Cheng et al. 2016; Gregory et al. 2022), and similar findings have been reported in several neurological populations (Trung et al. 2019; Tsai et al. 2020). Moreover, cognitive improvements may be more than transient, persisting beyond one-month post-treatment in some studies (Trung et al. 2019; Lang et al. 2020).

Despite the fact that cognitive deficits are highly prevalent in bipolar disorder, few TMS trials have focussed on cognitive improvement in this population. A recent review of this literature by Sciortino et al. (2021) revealed mixed findings, with evidence of both positive and negative findings. However, none of these previous studies employed iTBS, which as stated previously, may have enhanced potential to improve cognition.

In addition to modulating prefrontal cortical regions that underlie various cognitive executive abilities (Lowe et al. 2018), TMS delivered to the left DLPFC may also influence regions that are not directly targeted, such as the hippocampus, which is involved in memory and learning (Kim et al. 2019). For example, ipsilateral hippocampal volume increases have been reported after left DLPFC stimulation (Hayasaka et al. 2017; Noda et al. 2018), although the degree to which these changes impact cognitive functions is less clear (Furtado et al. 2013). Because bipolar disorder is characterised by reduced hippocampal volumes (Haukvik et al. 2022) and diminished memory functioning (Bourne et al. 2013), neurostimulation therapies that can also induce changes in medial temporal brain regions may be clinically beneficial (Ott et al. 2019).

The present study is the first to investigate potential cognitive improvements of iTBS in bipolar disorder, as well as to explore whether changes in cognitive functioning might be associated with iTBS induced increase in hippocampal volumes. The study was a secondary analysis of cognitive and hippocampal volume outcomes in a completed iTBS trial in bipolar depression that failed to show improvements in depressive symptoms (McGirr et al. 2021). This presented an opportunity to evaluate whether a course of iTBS delivered to the left DLPFC could improve cognition in the absence of confounding change in depressive symptoms. We hypothesised that relative to sham, iTBS treated patients would show improvements in cognitive, and particularly declarative memory functioning, and that these improvements would be associated with hippocampal volume increases.

## Materials and methods

The data for this secondary analysis was collected as a part of the iTBS trial for bipolar depression (McGirr et al. 2021), conducted in accordance with IFCN recommendations (Rossini et al. 2015). The study was conducted across two Canadian centres (University of British Columbia [UBC], British Columbia, and University of Calgary [UofC], Alberta) and the data collection spanned from October 2016 to March 2020. The original trial was registered with clinicaltrials.gov (NCT02749006) and was approved by UBC Clinical Research Ethics Board of the University of British Columbia and Conjoint Health Ethical Review Board of UofC. Written informed consent was obtained from all participants.

### Participants

Participants were recruited by referral from outpatient clinics and online and community advertisements. Inclusion criteria included: adult 18–70 years old; primary DSM-5 diagnosis of bipolar disorder Type I or II confirmed with the MINI International Neuropsychiatric Interview (Sheehan et al. 1998); experiencing a major depressive episode with score ≥18 on the 17-item Hamilton Depression Rating Scale (HDRS-17; Hamilton 1960); failure to achieve sufficient clinical response with at least one CANMAT (Yatham et al. 2018) first-line treatment for an acute bipolar major depressive episode. Participants were required to have been on a stable pharmacological regimen for two weeks prior to screening. Treatment was required to include a mood stabiliser (lithium >0.6mEq/L, valproate >350mM/L, or Lamotragine > 100mg), an atypical antipsychotic, or both. Exclusion criteria included: acute suicidality; current psychosis; substance use disorder within the past 3 months; seizure history; unstable medical condition; comorbid psychiatric condition deemed to be primary. Participants were also excluded if they had previous non-response to rTMS, current use of more than three antipsychotics, previous failure to respond to ECT in the current episode, or had initiated psychotherapy within the previous three months.

### Neurostimulation protocol

Eligible participants were enrolled consecutively and a stratified random number sequence was generated by an independent statistician for each site, according to treatment with mood stabilisers, atypical antipsychotics, or their combination. Randomisation occurred with allocation concealment *via* the envelope method to ensure that patients and investigators remained blind to treatment condition during the study.

Each site utilised a MagPro X100 stimulator (MagVenture, Denmark), with the UBC site utilising a dual active/sham Cool-B65 A/P coil and the UofC site a COOL-B70 or MCF-P-B70 placebo coil. Both sites utilised anatomical MRIs and neuronavigation (Visor2, ANT Neuro, the Netherlands). Resting motor threshold (rMT) was determined by visual inspection at the UBC site and by using EMG electrodes at the UofC site placed over the first dorsal interosseous muscle, with threshold determined as the stimulus intensity required to elicit 5/10 EMG responses >50µV.

Patients were randomised to sham- or active-iTBS, consisting of a total of 600 pulses per session delivered as triplets at 50 Hz repeated at 5 Hz (2s on 8s off) at 120% rMT, which had been shown to improve depressive symptoms in MDD (Blumberger et al. 2018). The left DLPFC was targeted using neuronavigation or the BeamF3 method (Beam et al. 2009) if participants could not tolerate an MRI (one participant). Daily treatments (five per week) were administered over four weeks.

### Neuropsychological assessment

Because the initial primary outcome measure of the trial was efficacy for depressive symptoms, patients were not pre-selected for demonstrating cognitive deficits. The International Society for Bipolar Disorders–Battery for Assessment of Neurocognition (ISBD-BANC) was used to assess cognitive functioning in patients in the areas of attention, processing speed, verbal and nonverbal learning/memory, working memory, and executive function (Yatham et al. 2010). The ISBD-BANC overlaps considerably with subtests comprising the MATRICS Consensus Cognitive Battery (MCCB; Nuechterlein et al. 2008), but further includes more complex verbal learning and executive function measures tailored to the cognitive impairments in BD.

The ISBD-BANC was administered by a research coordinator trained by the clinical neuropsychologist on the research team (IJT), both at baseline prior to neurostimulation, and at completion of the four-week trial. At follow-up, alternate forms for the California Verbal Learning Test – 2nd Edition (CVLT-2) (Delis et al. 2000) and Brief Visuospatial Memory Test- Revised version (BVMT-R) (Benedict 1997) were utilised. All test scores were converted to demographics-corrected T-scores. The following cognitive outcomes were used:

#### Primary outcome measure

*Global Cognition*: As in our previous pharmacological cognitive trial (Yatham et al. 2017), the primary measure was the global cognition score which is a composite score of the mean t-score value of the following measures: CVLT-II trial 1–5; CVLT-II delayed free recall; BVMT-R trial 1–3; BVMT-R delayed recall; Trail Making Test (Reitan and Wolfson 1985) trial A (TMT-A) time; TMT-B time; Continuous Performance Test- Identical Pairs (CPT-IP) trial 1–3; Animal Fluency; Letter-Number Sequencing; Spatial Span; Symbol Coding; Stroop (Golden and Freshwater 2002) Word; Stroop Colour; and Stroop Colour-Word.

##### Secondary outcome measures

*Verbal memory:* (mean t-score of the two CVLT-2 scores).

*Nonverbal memory:* (mean t-score of the two BVMT-R scores).

*Total memory:* (average of the Verbal and Nonverbal memory scores).

### Neuroimaging data acquisition and analysis

Three-dimensional T1-weighted MRI images were collected at baseline and four-week follow-up at the UBC MRI Research Centre on a Philips Achieva 3.0 T scanner and UofC on a GE 3.0 T MR750w scanner. The scanning parameters of the Philips Achieva scanner were: number of axial slices = 155; repetition time (TR) = 6.6 ms; echo time (TE) = 3.0 ms; flip angle (FA) = 8 degrees; field of view (FOV) = 240 mm × 240 mm × 155 mm; slice thickness = 1 mm. The scanning parameters of the GE scanner were: number of axial slices = 176; TR = 8.1 ms; TE = 3.2 ms; FA = 16 degrees; FOV = 256 mm × 256 mm × 176 mm; slice thickness = 1 mm.

The FreeSurfer image analysis suite (version 7.1.0, http://surfer.nmr.mgh.harvard.edu/) was used to segment the whole brain and the hippocampus from the T1-weighted images using a longitudinal pipeline. Specifically, data from two time points were longitudinally processed to generate an unbiased within-subject template, and all further processing steps were initialised using this template (Reuter et al. 2012). Hippocampal segmentation was carried out on the longitudinal processed output data using the longitudinal hippocampal segmentation tool in FreeSurfer (Iglesias et al. 2016), which uses a subject-specific atlas and treats all time points the same way to avoid processing bias, increasing the robustness of segmentation and measurement of subfield volumes. Total hippocampus volume was extracted for each hemisphere. Quality assurance involved visual inspection and outlier detection, defined as the volumes of more than three standard deviations of the mean. The FreeSurfer-derived measures of hippocampal and the total gray matter volumes were site harmonised using the longitudinal Combat method (Beer et al. 2020) implemented as per https://github.com/jcbeer/longCombat. This is a multistep method that progresses from data standardisation, to empirical Bayes estimation of scanner effects and linear mixed-effects models.

### Statistical analysis

Demographic and clinical variables were compared between groups using independent t-tests and chi-square tests. The analyses for the primary efficacy measure (global cognition) and secondary cognitive measures (verbal memory, nonverbal memory, total memory) were conducted using repeated measure ANOVA with time (baseline vs. follow-up) as a repeated measure and group (active vs. sham) as a between-subjects factor, with particular attention to the time by group interaction which would indicate differential change in cognitive performance between the two groups. Similar analyses were conducted for all individual test scores. In a supportive set of analyses we also used ANCOVA to evaluate cognitive group differences (active vs. sham) at follow-up after covarying for baseline cognitive score and change in mood symptoms (follow-up minus baseline MADRS and YMRS). For the cognitive analyses above, alpha values were adjusted based on the number of cognitive outcomes (alpha = .05/4 = .0125). The relationship between change in cognitive variables and change in mood symptoms was assessed using Spearman correlations. Time by group repeated measure analyses were also used to evaluate group differences in hippocampal volumes and in total brain grey matter volume, again with an emphasis on the group by time interactions. We evaluated the association between change in hippocampal volume and change in cognitive functioning in the active group using Pearson correlations, as well as partial correlations removing the effect of change in depressive symptoms.

## Results

### Group differences in demographic and clinical variables

Of 37 initial participants, two receiving active-iTBS and four receiving sham-iTBS dropped out of the study. One participant receiving active-iTBS completed three weeks of treatment before institutional COVID-19 closure required discontinuation; however, cognitive data was collected at the three week time point for this person and was included in the analyses. Overall, 31 participants (16 active, 15 sham) received cognitive testing, and their clinical and demographic characteristics are provided in Table 1. One person in the active group declined BVMT-R testing at baseline and follow-up, and therefore was missing nonverbal memory scores. Two patients in each group were left-handed. Patients in both groups were well matched demographically and clinically, with the exception that patients in the sham group had a higher number of prior depressive episodes than those in the active group. Additionally, although baseline YMRS scores were well within the euthymic range at follow-up for both groups, they were significantly higher in the sham group.

**Table 1.** Demographics and clinical characteristics of participants in the active and sham groups.^a^

Characteristics	Active (*N* = 16)		Sham (*N* = 15)		
Continuous variables	Mean	SD	Mean	SD	*p*
Age	46.4	13.6	48.1	11.4	.71
Education	15.4	3.6	15.4	2.2	.97
Premorbid-IQ	112.1	8.7	112.9	5.6	.79
YMRS^b^ – baseline	2.0	1.5	2.2	1.8	.73
YMRS^b^ – follow-up	1.1	1.6	2.3	1.3	.04*
MADRS^b^ – baseline	32.0	4.0	31.7	3.9	.85
MADRS^b^ – follow-up	24.5	10.8	22.0	10.0	.52
Illness length (years)^c^	19.2	14.3	23.1	10.5	.46
No. manic episodes	2.1	3.6	2.5	3.6	.73
No. depressive episodes	8.6	11.5	23.9	25.1	.04*
Categorical variables	*N*	%	*N*	%	*p*
Female	9	56.3	9	60.0	.83
Caucasian	16	100	15	100	.99
Mood Stabilizer	8	53.3	4	30.8	.28
Antipsychotic	1	6.7	3	23.1	.31
Mood Stabiliser and Antipsychotic	6	40	6	46.2	1.0

*Notes:* NAART active *n* = 12, sham *n* = 11; illness length active *n* = 14, sham *n* = 11; number manic episodes active *n* = 15, sham *n* = 13; medication data active *n* = 15, sham *n* = 13.

^a^MADRS = Montgomery Asberg Depression Rating Scale (10-item); Premorbid-IQ = North American Adult Reading Test full scale IQ; YMRS = Young Mania Rating Scale.

^b^Rating scales done at baseline.

^c^Illness length based on age at baseline minus age when first mood episode occurred (diagnosed manic or depressed episode).

*p < .05.

### Cognitive changes across groups

Baseline and follow-up cognitive data for active and sham groups, as well as the results from the repeated measure ANOVAs are summarised in Table 2. After correction for multiple comparisons there was a significant time effect for the global cognition score [F(1,29) = 12.1, *p* = .002]. There were no significant group effects (all *p* > .20) nor significant group by time interactions for the primary or any of the secondary cognitive outcome measures (Table 2; all *p* > .40). There were also no significant group by time interactions for any of the individual test scores that made up the global cognition score (all *p* > .05, data not shown).

**Table 2.** Repeated measure ANOVA results for cognitive variables and hippocampal volumes (mm^3^).^a^

	Active baseline		Sham baseline		Active follow-up		Sham follow-up		Group	Time	Group * time
Cognitive variables^b^	M	SD	M	SD	M	SD	M	SD	F	F	F
Global cognition	47.0	9.0	44.7	7.6	49.20	10.1	46.4	6.4	0.74	12.1**	0.21
Verbal memory	47.3	13.8	43.4	6.8	46.4	14.8	40.0	9.8	1.7	1.7	0.62
Nonverbal memory	54.7	12.2	51.1	12.5	57.6	10.1	53.8	9.3	0.93	4.9*	0.00
Total memory	50.1	12.5	47.2	8.4	50.8	13.2	46.9	8.5	0.81	0.04	0.22
Brain volumes^c^	M	SD	M	SD	M	SD	M	SD	F	F	F
Left hippocampus	3474	277	3506	539	3515	317	3465	520	0.00	0.00	3.9
Right hippocampus	3469	298	3498	574	3498	293	3541	558	0.04	4.2	0.14
Total hippocampus	6943	548	7005	1103	7013	581	7006	1060	0.01	1.7	1.6
Total brain gray	636,716	52,856	629,064	81,182	635,693	53,500	629,269	81,854	0.07	0.07	0.15

^a^Group = Group Effect; Time = Time Effect; Group * Time = Group by Time Interaction.

^b^Active *n* = 16, sham *n* = 15; Nonverbal Memory active *n* = 15.

^c^Active *n* = 12, sham *n* = 14.

**p < .01, *p < .05.

In the supportive ANCOVAs, there were similarly no significant differences between active and sham groups on any of the primary or secondary cognitive variables at follow-up after covarying for baseline cognition and change in mood symptoms (all *p* > 0.35).

There were no significant correlations between change in any of the cognitive variables and change in depression or mania symptoms, both within each group and when groups were combined (all *p*>.20).

### Hippocampal volume change across groups

Hippocampal data were available for 12 patients in the active group (3 had metal implants, 1 was not scanned due to COVID-19 closure) and 14 in the sham group (1 was claustrophobic). There was no significant difference in the proportion of patients from each site between the active and sham groups (X^2^ = .25, *p* = .62). The brain volume data are presented in Table 2 and visually in Supplementary Figure 1. There was a trend for a time effect for right hippocampal volume [F(1,24) = 4.2, *p* = .05], and a trend for a left hippocampal group by time interaction [F(1,24) = 3.9, *p* = .06]. No other significant group by time interactions were noted for other hippocampal volumes (all *p*>.20) or for total brain gray matter volume [F(1,24) = 0.15, *p* = .70].

### Correlations between cognitive change and hippocampal volume change

In the active group (*n* = 11), increase in left hippocampal volume was associated with improved nonverbal memory (*r* = .60, *p* = .05) (Supplementary Figure 2). This association remained unchanged after controlling for change in depressive symptoms (*r* = .62, *p* = .06). There were no other significant correlations between change in any hippocampal volumes and change in global cognition or memory variables (all *p* >.15).

## Discussion

The present study was a secondary analysis of an initial pilot trial investigating the efficacy of iTBS in bipolar depression, and the first to evaluate potential cognitive improvement with iTBS in bipolar disorder, as well as to explore whether cognitive changes were associated with increased hippocampal volumes. Overall, there was no evidence of cognitive improvement with iTBS delivered to the left DLPFC on global or specific cognitive domains. However, there was a non-significant trend for a modest increase in left hippocampal volume in the iTBS compared to the sham group, and this volume increase was associated with improved nonverbal memory functioning.

This study contributes to the mixed findings reported in studies investigating possible TMS-related cognitive improvements in bipolar disorder. Our results are in line with two prior TMS studies that also failed to observe cognitive improvements (Hu et al. 2016; Myczkowski et al. 2018), but at odds with two other studies reporting positive findings (Yang et al. 2019; McIntyre et al. 2021). Supplementary Table 3 provides a comparison of these studies with regard to several key patient, treatment, and methodological variables that could potentially influence findings (Miskowiak et al. 2017). With the exception of mood state, none of the variables in Supplementary Table 3 appear to clearly distinguish studies with positive cognitive findings from those with negative findings. Regarding mood state, all the studies that failed to observe cognitive improvements were conducted in acutely depressed patients, while the positive studies were conducted on patients who were symptomatically stable or remitted. It is possible that acute mood effects may influence baseline and/or follow-up cognitive performances in an unpredictable manner, such that the added ‘noise’ stemming from the cognitive effects of acute mood symptoms may confound the ability to detect TMS-related cognitive improvements if they are present. In contrast, in the studies investigating euthymic patients, it may be easier to detect treatment related effects in the absence of noise arising from acute mood symptomatology and associated cognitive variation.

Although we failed to observe cognitive improvements associated with iTBS, patients in the active group did exhibit a trend towards the expected finding of increased ipsilateral hippocampal volumes relative to the sham group. Hippocampal volume increases have also been observed in a previous iTBS trial in bipolar disorder (Baeken et al. 2020). Additionally, in the active group, left hippocampal volume increases were associated with improvements in nonverbal memory performance, and this occurred independent of change in mood state. Although it may have been expected that left hippocampal volume increases might be more closely associated with verbal rather than nonverbal memory changes, prior volumetric studies have also reported an association between left hippocampal volumes and nonverbal memory abilities (Shavitt et al. 2020). Overall, the presence of hippocampal volume increase in conjunction with improvement in memory functioning may reflect iTBS induced neuroplasticity within prefrontal-temporal lobe networks. Furthermore, our data suggest that such possible neuroplastic changes may be apparent only when structure and function are measured concurrently, as opposed to when only cognitive outcomes are assessed.

The current findings provide some support for the hypothesis that iTBS induces neuroplasticity within prefrontal-temporal networks. However, DLPFC stimulation alone may not be the most efficient and direct means of modulating hippocampal function. Previous studies in healthy participants reveal that by virtue of the strong connections between parietal cortex and medial temporal lobe regions, TMS of the former may serve as a form of ‘hippocampal-targeted’ TMS that induces strong effects on hippocampal based memory functions (Wang et al. 2014; Tambini et al. 2018). Furthermore, patients with major depression who received iTBS to DLPFC in conjunction with parietal stimulation augmentation demonstrated increased functional connectivity between hippocampus and DLPFC, increased hippocampal response, and a stronger correlation between hippocampal response and cognitive function relative to patients receiving DLPFC stimulation only (Mielacher et al. 2020). These findings suggest that in order to optimise modulation of hippocampal cognitive/memory functions, future iTBS studies in bipolar disorder should target these parietal cortical regions. Future neurostimulation studies in bipolar disorder should also aim to enhance the modulation of complex synaptic mechanisms such as metaplasticity, which is likely to be an important determinant underlying learning, memory, and other functions (Cantone et al. 2021). For example, modulation of metaplasticity may be achieved through the use of accelerated iTBS protocols at appropriately spaced intervals, with the aim of improving clinical outcomes (Thomson and Sack 2020).

Several strengths of this study included the utilisation of an iTBS TMS protocol, investigation of underlying hippocampal volumetric biomarkers of cognitive change, and assessment of multiple cognitive domains. However, several study limitations may have contributed to the negative cognitive findings reported herein. Although the sample size was modest, it was in line with the prior positive studies (Yang et al. 2019; McIntyre et al. 2021) that also assessed similar cognitive domains, arguing against the point that our study was clearly underpowered. Additionally, because our trial was initially designed to evaluate symptomatic rather than cognitive outcomes, we did not pre-select participants for cognitive impairment, and this may have contributed to possible ceiling effects that could have masked treatment-related cognitive improvements. This may have been particularly salient given that our participants were on average highly educated and showed above average intellectual functioning. Our randomisation was also stratified along the lines of medication use, and may have been different if the initial clinical trial was designed to target cognition. Nevertheless, the groups were well matched according to basic demographic and clinical variables such age, sex, and depressive severity, which decreases the likelihood that these variables served as confounds. A final limitation was the use of different coils and MRI scanners at the two sites; however, the fact that there was a comparable proportion of patients from each site in the active and sham groups minimises the likelihood of this potential confound. In keeping with recommendations for procognitive trials in bipolar disorder (Miskowiak et al. 2017), future studies investigating cognitive enhancing effects of iTBS should be sufficiently powered to detect clinically meaningful cognitive change, pre-select patients for cognitive impairments, and include participants in a euthymic mood state. Also, the use of parietal lobe or accelerated iTBS protocols may maximise the ability to promote memory and cognitive improvement in patients with bipolar disorder.

## Supplementary Material

Supplemental Material
