# Supplementary material for: Effects of intermittent theta-burst transcranial magnetic stimulation on cognition and hippocampal volumes in bipolar depression
Source: Dialogues Clin Neurosci. 2023 Mar 16;25(1):24–32. doi: 10.1080/19585969.2023.2186189 (PMC10026761; doi:10.1080/19585969.2023.2186189)
Supplement: Supplemental Material [file TDCN_A_2186189_SM1215.docx]

| **SUPPLEMENTARY TABLE 3. Key patient, treatment, and methodological variables across positive and negative studies** | | | | | |
| --- | --- | --- | --- | --- | --- |
|  | **Negative Studies** | | | **Positive Studies** | |
| **Study Variables** | **Torres, 2023** | **Hu, 2016** | **Myczkowski, 2019** | **Yang et al., 2019** | **McIntyre, 2021** |
| Diagnosis | Bipolar I, II | Bipolar II | Bipolar I, II | Bipolar I, II | Bipolar I, II |
| Symptom state | Depressed | Depressed | Depressed | Remitted | Remitted |
| Pre-screened cognitively | No | No | No | No | No |
| Sample size | Active n=16  Sham n=15 | Active left n=12  Active right n=13  Sham n=13 | Active n=20  Sham n=23 | Active n=25  Sham n=27 | Active n=16  Sham n=20 |
| Stimulation type | iTBS | High Frequency  Low Frequency | 18 Hz | High Frequency | High Frequency |
| Coil Type | Figure-8 | Figure-8 | H1 | Figure-8 | Figure-8 |
| Stimulation site | L DLPFC | L DLPFC  R DLPFC | L DLPFC | L DLPFC | L DLPFC |
| Treatment duration | 4 week | 4 week | 20 sessions | 2 week | 4 week |
| Cognitive domains | multiple | multiple/executive | multiple | multiple | multiple |
| iTBS= intermittent Theta Burst Stimulation; DLPFC= Dorsolateral Prefrontal Cortex | | | | | |


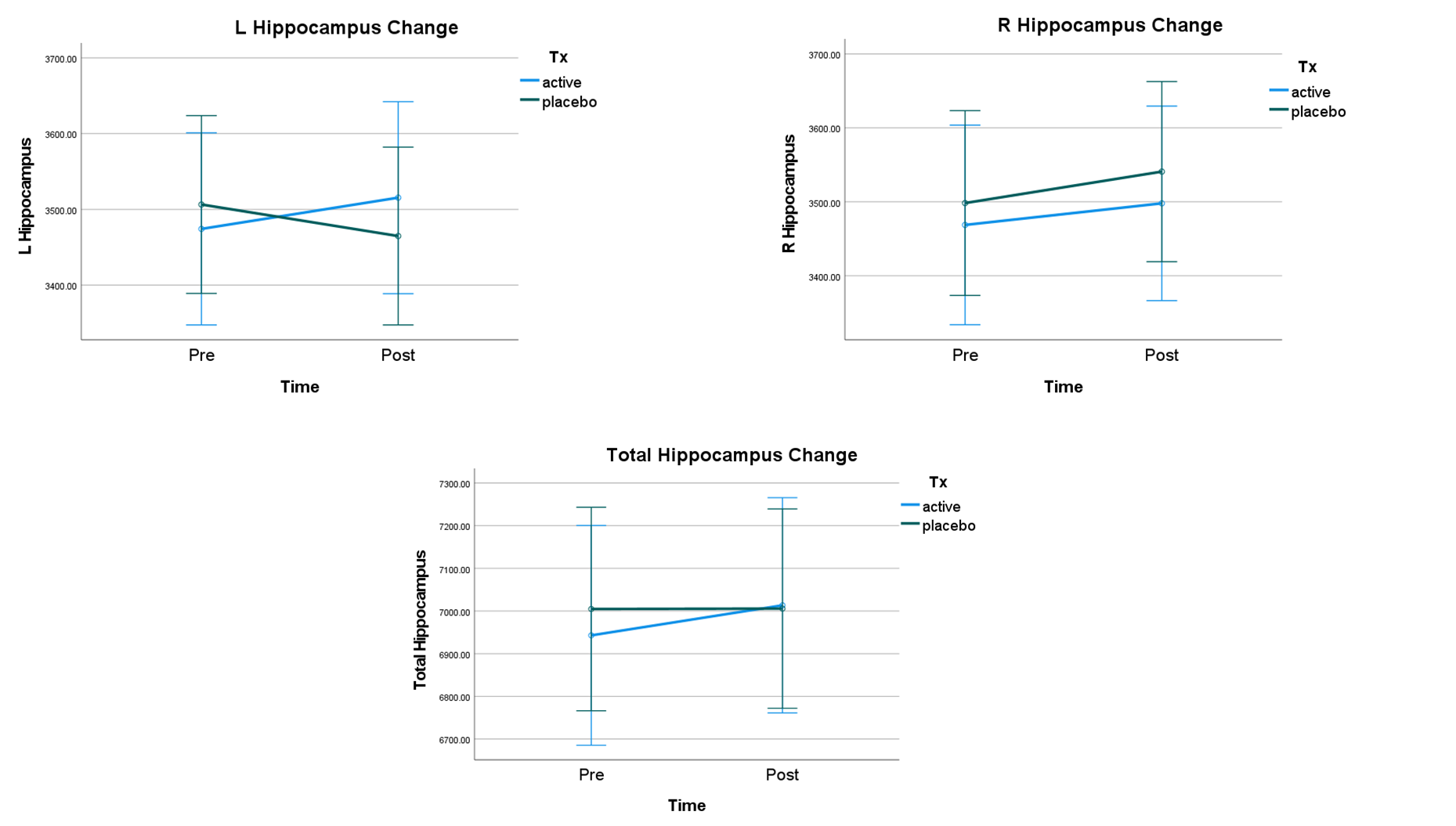


Supplementary Figure 1. Change in Hippocampal Volumes (mm^3^) in active-iTBS and sham iTBS. Error bars represent standard errors.


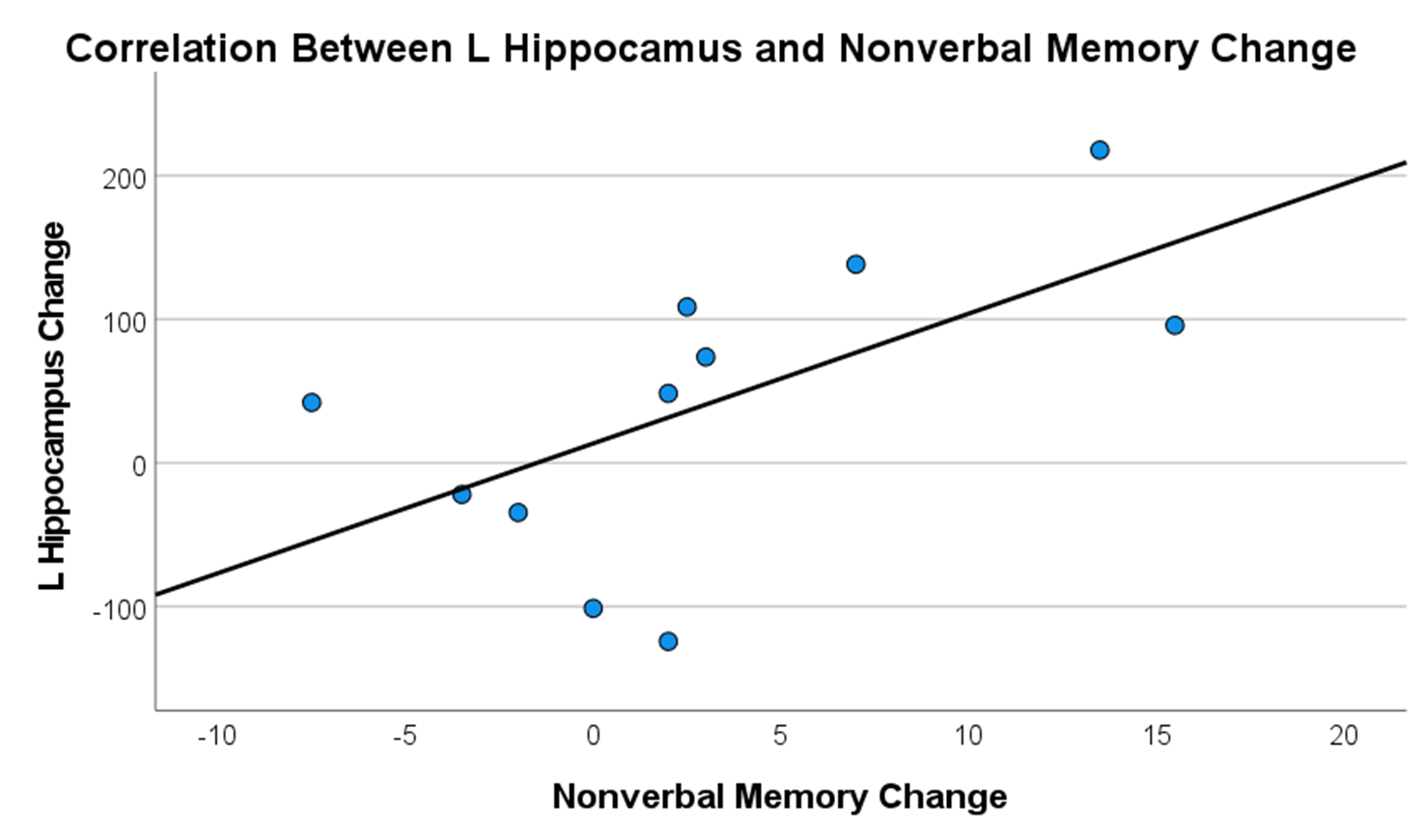


Supplementary Figure 2. Correlation Between Left Hippocampus Volume (mm^3^) and Nonverbal Memory Change
